# oxLDL and eLDL Induced Membrane Microdomains in Human Macrophages

**DOI:** 10.1371/journal.pone.0166798

**Published:** 2016-11-21

**Authors:** Stefan Wallner, Margot Grandl, Gerhard Liebisch, Markus Peer, Evelyn Orsó, Alexander Sigrüner, Andrzej Sobota, Gerd Schmitz

**Affiliations:** 1 Institute for Clinical Chemistry and Laboratory Medicine, University Hospital Regensburg, Regensburg, Germany; 2 Department of Cell Biology, Nencki Institute of Experimental Biology, Warsaw, Poland; Medical University of South Carolina, UNITED STATES

## Abstract

**Background:**

Extravasation of macrophages and formation of lipid-laden foam cells are key events in the development and progression of atherosclerosis. The degradation of atherogenic lipoproteins subsequently leads to alterations in cellular lipid metabolism that influence inflammatory signaling. Especially sphingolipids and ceramides are known to be involved in these processes. We therefore analyzed monocyte derived macrophages during differentiation and after loading with enzymatically (eLDL) and oxidatively (oxLDL) modified low-density lipoproteins (LDL).

**Methods:**

Primary human monocytes were isolated from healthy, normolipidemic blood donors using leukapheresis and counterflow elutriation. On the fourth day of MCSF-induced differentiation eLDL (40 μg/ml) or oxLDL (80 μg/ml) were added for 48h. Lipid species were analyzed by quantitative tandem mass spectrometry. Taqman qPCR was performed to investigate transcriptional changes in enzymes involved in sphingolipid metabolism. Furthermore, membrane lipids were studied using flow cytometry and confocal microscopy.

**Results:**

MCSF dependent phagocytic differentiation of blood monocytes had only minor effects on the sphingolipid composition. Levels of total sphingomyelin and total ceramide remained unchanged, while lactosylceramides, cholesterylesters and free cholesterol decreased. At the species level most ceramide species showed a reduction upon phagocytic differentiation. Loading with eLDL preferentially increased cellular cholesterol while loading with oxLDL increased cellular ceramide content. Activation of the salvage pathway with a higher mRNA expression of acid and neutral sphingomyelinase, neutral sphingomyelinase activation associated factor and glucosylceramidase as well as increased surface expression of SMPD1 were identified as potentially underlying mechanisms. Moreover, flow-cytometric analysis revealed a higher cell-surface-expression of ceramide, lactosylceramide (CDw17), globotriaosylceramide (CD77), dodecasaccharide-ceramide (CD65s) and GM1 ganglioside upon oxLDL loading. ApoE in contrast to apoA-I preferentially bound to the ceramide enriched surfaces of oxLDL loaded cells. Confocal microscopy showed a co-localization of acid sphingomyelinase with ceramide rich membrane microdomains.

**Conclusion:**

eLDL leads to the formation of lipid droplets and preferentially induces cholesterol/sphingomyelin rich membrane microdomains while oxLDL promotes the development of cholesterol/ceramide rich microdomains via activation of the salvage pathway.

## Introduction

Atherosclerosis represents a leading cause of death in industrialized countries [1, 2]. It is initiated by the enhanced perfusion and retention of cholesterol-rich, apoB-containing lipoproteins in the vessel wall [3]. These lipoproteins can be chemically modified by oxidation or enzymatic cleavage increasing their pro-inflammatory and atherogenic potential. They are cleared by extravasated monocyte-derived macrophages which, due to limited negative feedback, lead to expansion of the phagosome and transform into lipid laden foam cells that contain a large quantity of lipid droplets [4, 5]. Macrophages are in addition able to export excess cholesterol to extracellular acceptors such as high-density lipoprotein (HDL) particles in reverse cholesterol transport. For this purpose they utilize the major HDL apolipoprotein apoA-I and the ABC-transporters ABCA1 and ABCG1 as well as the scavenger receptor B1 (SR-BI) [6].

In the current study we used enzymatically modified low-density lipoproteins (eLDL) [7] and oxidized low-density lipoproteins (oxLDL) [8, 9] to specifically mimic the properties of *in vivo* occurring lipoprotein modifications. eLDL is characterized by proteolytic cleavage of apoB and hydrolysis of core cholesteryl esters, leading to liposome-like, coreless LDL particles, rich in unesterified cholesterol, free fatty acids and lysophospholipids. These particles resemble lesion derived LDL present at early stages in atherosclerotic lesions and induce storage of cholesteryl esters and triglycerides in lipid droplets leading to the “foamy” phenotype of macrophages [10]. In contrast, oxLDL particles are similar to polar surface modified particles found in lesions [11]. Oxidation of LDL renders oxLDL resistant to lysosomal hydrolysis and traps partially hydrolyzed oxLDL within the endolysosomal compartment [12], leading to phospholipidosis [5, 13] and impaired release of cholesterol from lysosomes [14, 15]. The differential composition and effects of eLDL and oxLDL have been reported recently [5, 10].

In the macrophage membrane, cholesterol loading leads to a deregulation of membrane homeostasis inducing an altered membrane composition, which is a major event in atherosclerotic progression [16]. Intracellular lipid flux and membrane lipid composition are essential components of inflammatory signaling. Especially lipid rafts represent highly dynamic membrane microdomains that play an essential role in transmembrane signaling. They modulate the compartmentalization of signal transduction by facilitating or inhibiting the assembly of signaling complexes [17]. Modest changes in their lipid composition are sufficient to induce membrane and protein reorganization with subsequent signal transduction. The physiological relevance of this process in immune cells could be shown for a variety of receptors and processes. For example in macrophages the recruitment of TNF receptor 1 to lipid rafts is essential for NF-kappa B activation [18]. Lipid rafts contain two to three-fold higher amounts of cholesterol in comparison to the surrounding membrane and are also strongly enriched in sphingolipids and glycosphingolipids such as gangliosides [19]. Especially sphingomyelin that acts as a receptor for cholesterol is known to be essential for lipid raft formation and membrane functionality [20].

Sphingolipids are of special importance because they also influence the formation of atherosclerotic lesions by mediating lipoprotein uptake [21] and serving as precursors for signaling lipids [22]. They are ubiquitous and structurally diverse, based upon a common sphingobase backbone which can be N-acetylated to form ceramides (Cer). Further head group modifications lead to diverse glycosphingolipid and sphingomyelin (SM) species. Sphingomyelin is a major plasma membrane constituent that can strongly interact with unesterified cholesterol in animal cells. It can also serve as a cellular source for choline groups [23]. Ceramides can act as cellular mediators, regulating proliferative and apoptotic processes in the ceramide/sphingosine-1-phosphate rheostat via diverse signaling pathways including G-protein coupled receptor signaling [24, 25]. Ceramide biosynthesis is dependent either on *de novo* synthesis from palmitoyl CoA via sphinganine or sphingobase liberation from sphingosine-1-phosphate through the salvage pathway [26, 27]. Sphingomyelinase dependent conversion of sphingomyelin to ceramides is regarded as a starting signal for apoE mediated tissue remodeling of the damaged vasculature and in nerve injury [28–30].

In previous studies we examined lipid remodeling during phagocytic differentiation and compared *in vitro* lipoprotein loading of the two prototypic lipoproteins eLDL or oxLDL with unmodified LDL using lipidomics, transcriptomics, flow cytometry and fluorescent high-content imaging [31–35]. We could especially show that oxLDL increased ceramide and lactosylceramide expression leading to ceramide-rich membrane microdomains, whereas loading with eLDL induced sphingomyelin/cholesterol-rich membrane microdomains [13]. In the current study we therefore analyze these effects further in detail and look at underlying mechanisms as well as on membrane microdomains sphingolipid composition during differentiation, lipid loading and HDL_3_ mediated deloading.

## Materials and Methods

### Chemical and enzymatic modification of low density lipoproteins

Isolation of human native LDL (1.006 mg/mL<d<1.063 mg/mL) from plasma of healthy blood donors and subsequent enzymatic degradation was performed as described previously [36] with slight modifications. Briefly, LDL was diluted to 2 mg/mL protein in PBS (w/o Ca^2+^; Mg^2+^). Enzyme treatment (eLDL) was conducted with trypsin (6.6 μg/mL) (Sigma, Germany) and cholesterol esterase (40 μg/mL) (Seikagaku, Japan) for 48 h at 37°C. Oxidative modification (oxLDL) of LDL was performed according to published protocols [37] by dialyzing LDL (1 mg of protein per mL) against 5 μM CuSO_4_. Modified lipoproteins were stored at 4°C and used within a week. During LDL preparation and subsequent modification, general precautions were taken to avoid lipopolysaccharide (LPS) contamination.

### Monocyte isolation, differentiation and lipid loading

Primary human monocytes were obtained from healthy normolipidemic volunteers with the apolipoprotein E3/E3 genotype by leukapheresis and counterflow elutriation as described previously [38]. The study was approved by the ethics committee of the University Hospital Regensburg and donors gave their written consent (Universitätsklinikum Regensburg, Ethikkommission der medizinischen Fakultät, proposal 08/119). Monocyte cultivation and differentiation was conducted according to Stoehr et al. [39]. Briefly, elutriated monocytes were seeded at 1x10^6^ cells/mL in macrophage serum-free medium (Invitrogen, Germany) supplemented with 50 ng/mL recombinant human monocyte-colony stimulating factor (MCSF; R&D Systems, USA) to induce phagocytic differentiation. Cells were cultured either on plastic petri dishes (Sarstedt, USA), Ultra Low Attachment 6-well plates (Costar Corning, Germany), Cell+ 6-well plates (Sarstedt, USA) or Lab-Tek II glass chamber slides (NalgeneNunc Intl., USA) and incubated at 37°C/5% CO_2_.

**Fig 1:**
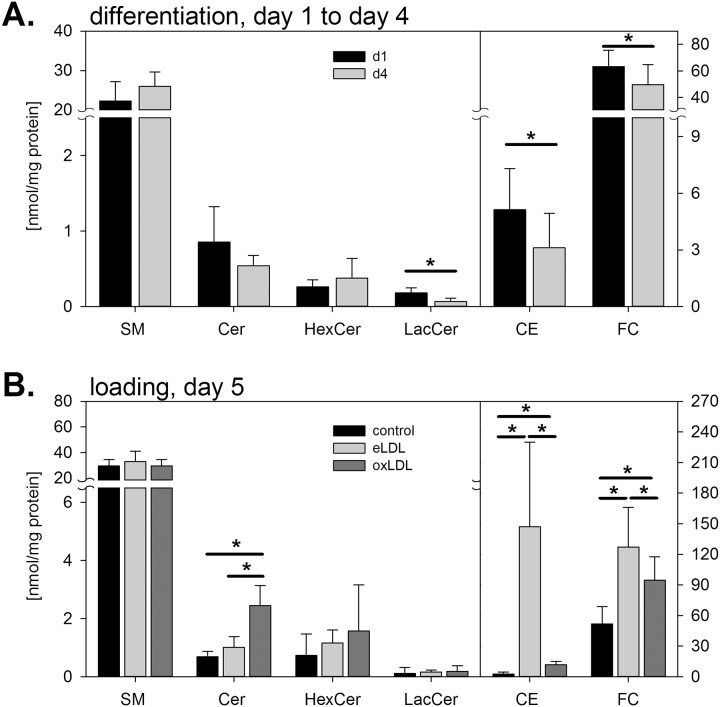
Quantitative mass spectrometric analysis of lipid classes. Lipid species composition of human blood monocytes was analyzed **(A)** on day one (d1) and four (d4) after MCSF differentiation and **(B)** following 24h enzymatically modified (eLDL) or oxidized (oxLDL) low density lipoprotein loading. Lipid classes include sphingomyelin (SM), ceramides (Cer), hexosylceramides (HexCer), lactosylceramides (LacCer), cholesteryl ester species (CE) and free cholesterol (FC). mean +/- SD. * = p< 0.05. n = 6.

On the fourth day loading with eLDL (40 μg/mL) or oxLDL (80 μg/mL) was performed for either 24h in the lipidomic experiments (Fig 1 and S1 Fig) or for 48 h in all other experiments. Cells were harvested on day(s) one (initial state), four (MCSF differentiated) and six (loaded) respectively and analysis was performed. Cells were checked for apoptosis by flow cytometry using AnnexinV-FITC and propidium iodide (PI) staining.

For HDL_3_ deloading, cells were first differentiated and incubated with LDL, eLDL or oxLDL as described previously. On day six, cells were washed and incubated with serum free medium containing MCSF and incubated another 24 h with HDL_3_ (100 μg/mL) as described previously [40]. Cell harvest and analysis was performed as described previously for MCSF-differentiation and lipoprotein loading.

### Lipid analysis

Lipid extraction at desired time points was performed by the method of Bligh and Dyer [41] or by butanolic extraction as described previously [42]. Mass spectrometry based targeted lipid analysis in the presence of isotopic labeled or not naturally occurring lipid species as internal standards was performed essentially as described previously [31, 42–46]. In brief, cells were washed twice in PBS, lysed in 0.1% SDS, sonicated and aliquots corresponding to each 100 μg total protein (BCA assay, Pierce, USA) were used for lipid extraction.

Direct flow injection utilized a 1100 series binary pump (Agilent, Germany) coupled via electrospray ionization (ESI) to a Quattro Ultima tandem mass spectrometer (Micromass, UK). HILIC LC-ESI-MS/MS was conducted using a 1200 series binary pump (Agilent, Germany) and a hybrid triple quadrupole linear ion trap mass spectrometer API 4000 Q-Trap (AB Sciex, Germany). Quantification was performed by standard addition calibration to cell homogenates using a number of naturally occurring lipid species for each lipid class. Lipid nomenclature was applied according to the notation proposed by Liebisch et al [47].

### Quantitative PCR

Total RNA of harvested cells was isolated by RNeasy Mini Kits (Qiagen, Germany) and DNA digestion was performed on column. Integrity of the extracted RNA was verified on an Agilent 2100 bioanalyzer equipped with an RNA 6000 Nano LabChip reagent set (Agilent, Germany). Extracted RNA was quantified with a Nanodrop ND-1000 photometer (PeqLab, Germany). qPCR was performed using commercially available, predesigned TaqMan^®^ PCR assays on the ABI Prism 7900 HT Sequence Detection System (Perkin Elmer-Applied Biosystems, Germany) according to manufacturer’s instructions. For quantification we used the standard curve method. Therefore a stock of total RNA from macrophages was serially diluted and a standard curve with 50, 25, 12.5, 6.25 and 3.125 ng total RNA was generated for all to determine the relative expression of genes and 18s mRNA in each sample. Finally the results were normalized to the endogenous control 18sRNA. All experiments were performed in triplicates.

### Western blotting

Antibodies against ABCA1 (clone: AB.H10, mouse), ABCG1 (synthetic peptides AA185-196 and AA347-362 against an internal region of human ABCG1/NP_058198, rabbit) or PLTP (mouse) were purchased from Abcam (UK), Pineda (Germany) or were a kind gift from M. Jauhiainen (National Public Health Institute, Department of Molecular Medicine, Helsinki, Finland), respectively. Cells were washed with PBS, frozen (-20°C), and thawed twice. Cell debris was separated by centrifugation at 10.000 x g for 10 min at 4°C. Proteins were separated by SDS-PAGE and transferred to PVDF (polyvinylindene fluoride) membranes. For detection of some proteins non reducing condition were necessary. Incubations with antibodies were performed in 5% nonfat dry milk in PBS, 0.1% Tween. Detection of the proteins was carried out with the ECL Western blot detection system and following quantification with the lumi-imager.

### Staining of ceramide and acid sphingomyelinase

Cells cultured on coverslips were washed with PBS, fixed in 4% paraformaldehyde (15 min), quenched with 50 mM NH_4_Cl/glycine (15 min) and blocked with PBS/0.5% BSA (15 min) at 20°C. Afterwards, cells were incubated with anti-ceramide mAB (MID15B4, Alexis, Germany) for 4 h and labeled with anti-mouse Cy5 (Abcam, UK) for 1 h at 4°C, followed by incubation with theta-toxin-FITC for 20 min at 4°C (Competence center for fluorescence biology KFB, Regensburg, Germany) for cholesterol staining. SMPD1 staining was performed by incubation of fixed cells for 45 min with rabbit polyclonal anti-SMPD1 antibody (Santa Cruz Biotechnology, USA) at 2 μg/mL. Cells were then washed in PBS-BSA and stained for 45 min with 200 ng/mL 488 Alexa goat anti-rabbit antibody (Molecular Probes, Netherlands). Nonspecific fluorescence was excluded by performing controls without primary antibody. Control staining was performed without primary antibody. For sphingomyelin staining, cells were incubated with lysenin-His for 3 h at 4°C followed by anti-His probe (H-15, Santa Cruz Biotechnology, USA) incubation for 2 h and subsequent incubation with anti-rabbit Texas Red for 30 min (Calbiochem, Germany).

Lysenin gene supplemented with His-tag at the N-terminus was synthesized and subcloned into pT7-7 plasmid and the recombinant protein was produced in *E*. *coli* [48]. All stainings were performed at 4°C. Images were acquired with an inverted TCS-4D confocal microscope (Leica, Germany) equipped with an Ar/Kr 75 mV mixed-ion laser (MellesGriot/Omnichrome, USA) and observed with an oil immersion objective 100 x lens. Acquisition was performed with the Scan Ware software package of the instrument and analysis was done using the software Metamorph (Universal Imaging Corp., USA). Fluorescence images were acquired with a 488 nm laser line (excitation) detected with a 535/405 nm band pass filter (emission) and observed with an oil immersion objective Plan Apo 100x lens (NA 1.3).

### Determination of cell surface ceramide

Cells were harvested gently from non-adherent dishes, washed with PBS (w/o Ca^2+^; Mg^2+^) and resuspended in PBS (w/o Ca^2+^; Mg^2+^)/0.5% BSA to yield 5x10^6^ cells/mL. Flow-cytometric analysis of surface exposed ceramide of 5x10^5^ cells was performed. Cells were incubated for 15 minutes on ice with saturating concentrations of the antibodies against ceramide (MID15B4, Alexis), CDw17 (G035-FITC, Becton-Dickinson), CD65s (VIM2-FITC, Caltag) and CD77 (38–13, Immunotech). Monoclonal antibodies used for detection were directly fluorochrome-conjugated or indirectly labeled by subsequent incubation with fluorochrome-conjugated secondary reagents (ceramide: goat anti mouse-FITC, Becton-Dickinson; CD77: goat anti rat-PE, Dianova) after two washing steps with PBS containing 0.5% BSA. GM1 was stained using Alexa Fluor 488 labeled cholera toxin subunit B (Molecular Probes).

### Detergent lysis and flotation gradient

Cells were harvested at the differentiated and eLDL and oxLDL loaded states, crude membrane fraction was isolated and Lubrol-WX detergent resistant microdomains were prepared as described previously [49]. These fractions were analyzed by ESI-MS/MS.

### ApoE and apoA-I binding and uptake assay

After washing the cells with PBS, they were resuspended in PBS/0.5% BSA at 5 x 10^6^/ml and incubated with 10μg/ml of apoE-Cy3 and apoA-I-Cy5 at 4°C and at 37°C for 5 min. Then cells were washed three times with cold PBS/0.5% BSA and analyzed by flow cytometry.

Cells cultured on coverslips were washed with PBS and incubated with 10μg/ml of apoE-Cy3 and apoA-I-Cy5 at 4°C to 5 min and washed with cold PBS. Subsequently cells were fixed using 4% paraformaldehyde in PBS for 15 min on ice. Afterwards cells were washed with cold PBS and analyzed by confocal microscopy.

### STED microscopy

Stimulated emission depletion (STED) microscopy was performed in cooperation with PD Dr. Harke and Prof. Dr. Hell at the Max Planck Institute for Biophysical Chemistry in Göttingen, Germany. A detailed description of the method can be found in [50].

### Statistical analysis

Statistical analysis was performed in SPSS 21.0. Paired t-tests were used to identify significant changes between days 1 and day 4 of monocyte to macrophage differentiation. In these analyses no correction for multiple testing was considered necessary. In order to test for statistical significance between day 6 control, eLDL and oxLDL loaded cells repeated measure ANOVA with pairwise and a LSD corrected post-hoc comparison was conducted. For total HexCer species Greenhouse—Geisser correction was additionally applied because sphericity was violated according to Mauchly’s test. Results are expressed as mean ± SD. Sigma Plot 12.5 and Excel 2010 were used for visualization.

## Results

### Transcriptomics shows increases in sphingolipid catabolic and decreases in sphingolipid biosynthetic transcripts during differentiation

While levels of total sphingomyelin (SM), ceramide (Cer) and hexosyl ceramide (HexCer) were not significantly altered during differentiation, total amounts of lactosyl ceramide (LacCer) and cholesteryl ester (CE) species as well as free cholesterol (FC) were significantly reduced (n = 6) (Fig 1A). Moreover, individual lipid species within sphingolipid classes were also significantly altered with increasing levels of SM 14:0, 24:3 and 24:2 species, while SM 15:0, 16:1, 18:0 and 22:0 decreased (S1A–S1D Fig). Concomitantly, Cer d18:1/18:0, 20:0, 22:0, 23:0 and 24:0 decreased, while HexCer d18:1/16:0 increased and LacCer 16:0, 22:0, 24:0 and 24:1 decreased during MCSF-dependent phagocytic differentiation.

**Fig 2:**
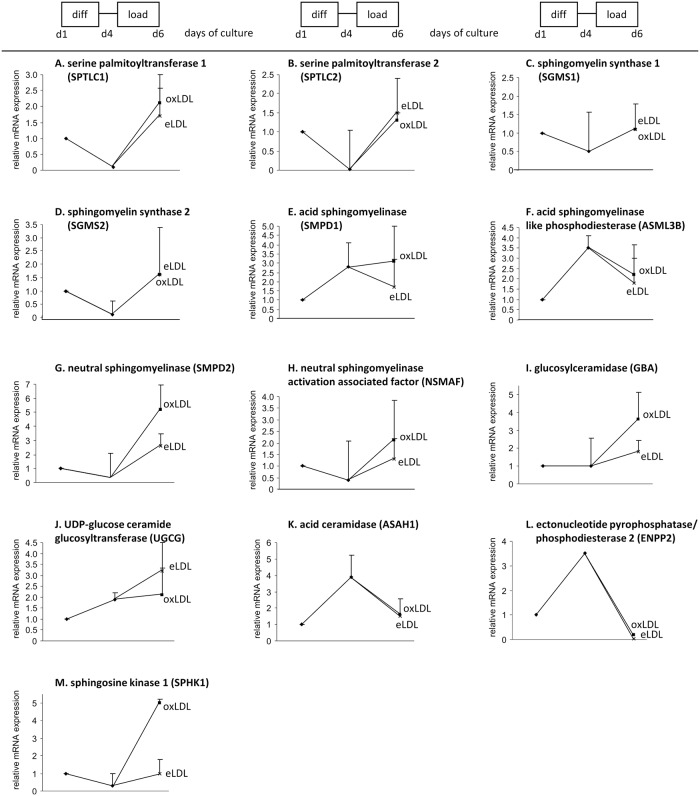
Taqman RT-PCR analysis of enzymes mainly involved in sphingolipid biosynthesis and metabolism. Gene expression was monitored of 4 day differentiated macrophages and of 48 hours eLDL and oxLDL loaded macrophages (day 4 to day 6. RT-PCR was standardized to 18s rRNA as a reference. mean + SD. n = 3.

During four days of MCSF-dependent monocyte/macrophage differentiation, mRNA expression of serine palmitoyltransferases 1+2 (SPTLC1+2), which are involved in ceramide de-novo synthesis as well as the transcriptional levels of sphingomyelin synthases, 1 and 2 which convert ceramides into sphingomyelin were downregulated (Fig 2A–2D). Analysis of the sphingomyelinases, which convert sphingomyelin to ceramide, revealed an upregulation of mRNA expression of acid sphingomyelinase (SMPD1) (2.8-fold) and acid sphingomyelinase like phosphodiesterase (ASML3B) (3.5-fold) (Fig 2E and 2F) whereas the mRNA expression of neutral sphingomyelinase (SMPD2) and neutral sphingomyelinase activation associated factor (NSMAF) was downregulated 1.5-fold during differentiation (Fig 2G and 2H). mRNA expression of glucosylceramidase (glucosidase beta acid, GBA) involved in the degradation of glucosylceramide into ceramide did not change during differentiation (Fig 2I). UDP-glucose ceramide glucosyltransferase (UGCG) the enzyme that converts ceramide to glucosylceramide and acid ceramidase (ASAH1) degrading ceramide to sphingosine showed an upregulation of mRNA expression (2 and 3.9-fold respectively) during differentiation (Fig 2J and 2K). The mRNA expression of sphingosine kinase 1 (SPHK1) which generates sphingosine-1-phosphate (S1P) from sphingosine was downregulated 1.5-fold (Fig 2M). In summary, upregulation of sphingolipid catabolizing enzymes on the one hand (ASAH1, SMPD1, and ASML3B) and downregulation of sphingolipid synthesis pathway enzymes (SGMS1 and SGMS2) point towards reduced sphingolipid biosynthesis.

Taqman qPCR analysis and western blotting additionally showed a strong upregulation of the lipid transporters ABCA1 and ABCG1 during MCSF-dependent phagocytic differentiation (S2 Fig).

### Loading with oxLDL increases cellular ceramides

While total sphingomyelin levels remained almost constant after loading with modified lipoproteins (Fig 1B), several individual sphingolipid species changed significantly with a similar pattern. Loading with eLDL significantly elevated sphingomyelin SM 15:0, 16:1, 18:0, 18:1, 20:1, 22:0, 22:2, 24:0 and 24:2 levels and oxLDL loading significantly increased mainly species containing saturated acyl-residues SM 14:0, 15:0, 16:1, 18:0, 18:1, 22:0, 24:0, 24:2 levels (S1E Fig).

Interestingly, only oxLDL led to a strong and significant increase of total ceramides as well as of multiple ceramide species (Cer d18:1/16:0, 18:0, 22:0, 23:0, 24:0, and 24:1, respectively), ranging between 3 to 5-fold, compared to control (S1F Fig). In contrast, eLDL significantly increased only Cer 18:1/22:0, 23:0 and 24:0. The most prominent elevations in ceramide species after lipoprotein loading were found in Cer d18:1/16:0 >24:1 > 24:0 > 22:0 > 23:0.

In addition, both eLDL (63-fold) and oxLDL (5-fold) significantly elevated most detectable cholesterylester species levels compared to control (Fig 1B and S1H Fig), resulting in an average total cholesterylester content of 147 nmol/mg for eLDL and 12 nmol/mg for oxLDL, respectively. Thus, eLDL loading was able to induce massive increases in cholesterylester species, while in oxLDL loaded cells the elevations were substantially lower but still significant.

### Transcripts for the ceramide salvage pathway are upregulated by oxLDL

Ceramides are generated either by hydrolysis of sphingomyelin or reconversion of complex sphingolipids via sphingosine (salvage pathway) or *de novo* synthesized from palmitoyl-CoA via sphinganine and dihydroceramide using ceramide synthases and desaturases (S3 Fig). Acid and neutral sphingomyelinases are the enzymes that degrade sphingomyelin to ceramide within the salvage pathway. To elucidate the underlying reason for the ceramide elevation during oxLDL loading, TaqMan real-time RT-PCR was performed for the enzymes involved in ceramide generation and degradation in MCSF differentiated, as well as eLDL and oxLDL loaded macrophages.

During eLDL loading serine palmitoyltransferases 1 and 2 (SPTLC1, SPTLC2) were 1.7 and 1.5-fold and during oxLDL loading 2.1 and 1.3-fold upregulated (Fig 2A and 2B). Sphingomyelin synthase 1 was 1.1-fold and sphingomyelin synthase 2 was 1.6-fold upregulated during lipid loading independent of the lipoprotein (Fig 2C and 2D). SMPD1 and ASML3B mRNA expression was 1.7 and 1.8-fold downregulated during eLDL loading. oxLDL loading however revealed a 3.1-fold upregulation of SMPD1 and a 2.3-fold downregulation of ASML3B compared to MCSF. mRNA expression of SMPD2 and NSMAF showed a stronger upregulation during oxLDL loading (5.2- and 2.2-fold) than during eLDL loading (2.7- and 1.3-fold) (Fig 2G and 2H). The stronger upregulation of the sphingomyelinases SMPD1, SMPD2 and NSMAF during oxLDL loading compared with eLDL loading fits well with the oxLDL induced ceramide elevation. GBA was upregulated more strongly during oxLDL than during eLDL loading (3.7- vs 1.8-fold) (Fig 2I) also reflecting increased ceramide formation during oxLDL compared to eLDL loading. UGCG was higher upregulated during eLDL than during oxLDL loading (3.2- vs 2.2-fold) (Fig 2J) while SPHK1 was stronger upregulated during oxLDL than during eLDL loading (5- vs 1.2-fold) (Fig 2M) although no significant differences in sphingosine content were observable (S4 Fig).

No significant changes during eLDL and oxLDL loading could be found in the mRNA expression of serine palmitoyltransferase 1+2 (SPTLC1+2), sphingomyelin synthase, 1 and 2, N-acylsphingosine amidohydrolase 1 (ASAH1; acid ceramidase), sphingosine kinase 2 (SPHK2) and ectonucleotide pyrophosphate/phosphodiesterase 2 (ENPP2, autotaxin). The mRNA expression of alkaline ceramidase and non-lysosomal ceramidase 2 (neutral ceramidase) was below the detection level.

### Ceramides on the cell surface increase upon oxLDL loading

**Fig 3:**
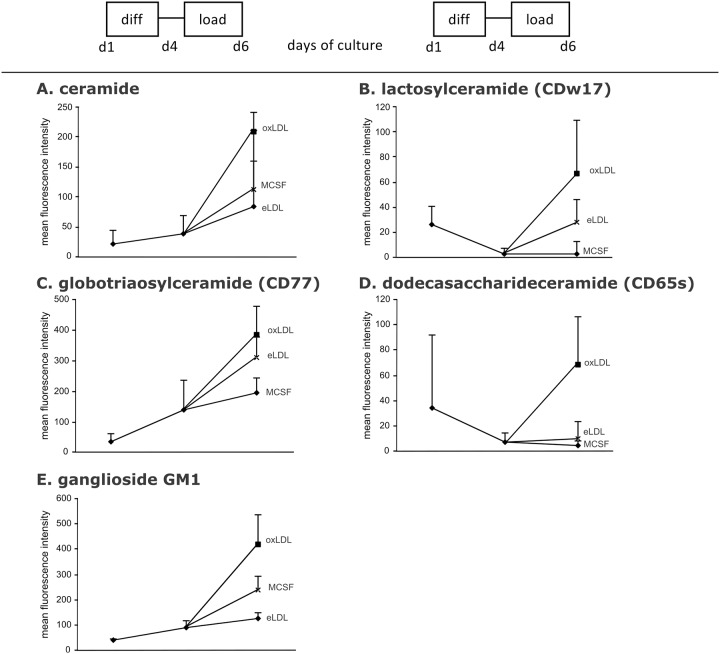
Cell surface expression of ceramide and complex sphingolipids. Depicted is the mean fluorescence intensity as analyzed by flow cytometry using monoclonal antibodies for ceramide, lactosylceramide globotriaosylceramide and dodecasaccharideceramide, and cholera toxin subunit B for ganglioside GM1 after MCSF differentiation (d4) and lipoprotein loading (d6) with eLDL, oxLDL or control (MCSF) **(A)** ceramide, **(B)** lactosylceramide/CDw17, **(C)** globotriaosylceramide, **(D)** dodecasaccharideceramide and **(E)** GM1 ganglioside. n = 3. Data on surface ceramide and lactosylceramide have been published previously [13].

To investigate whether these adaptions also modulate ceramide and complex sphingolipid composition on the cell surface, flow cytometry was performed (Fig 3). During MCSF induced differentiation, surface quantities of ceramide, globotriaosylceramide/CD77 and GM1 ganglioside increased, while the amount of surface associated lactosylceramide/CDw17 and dodecasaccharideceramide/CD65s decreased.

Loading with oxLDL led to a strong increase in exposed surface ceramides and complex sphingolipids compared to MCSF differentiated macrophages while loading with eLDL only had a minor effect. These results indicate that oxLDL, in comparison to eLDL, not only increased total cellular ceramide content through the salvage pathway but also induced an increase in cell surface ceramides and complex sphingolipids. Ceramide-backbone glycosphingolipid surface expression was increased to a higher extent after oxLDL compared to eLDL loading.

### oxLDL loading leads to higher SMPD1 levels on the cell surface

**Fig 4:**
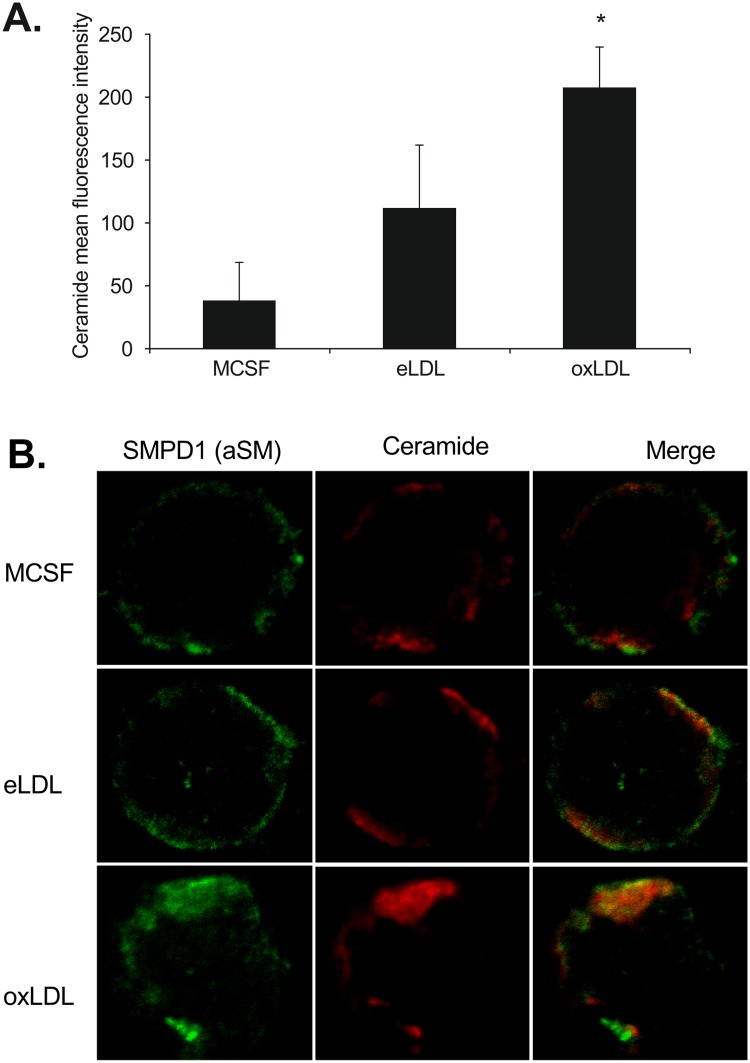
oxLDL induces cell surface ceramides and SMPD1 co-localization at the membrane. **(A)** Mean fluorescence intensity of MID15B4 labeled ceramide in MCSF treated, eLDL or oxLDL loaded macrophages. p < 0.05. **(B)** SMPD1 (aSM) surface expression (green) and cell surface ceramide content and distribution (red) after MCSF differentiation, eLDL or oxLDL loading. Merge: orange. Representative cells are shown.

As mentioned above, qPCR analysis showed an upregulation in the transcription of acid sphingomyelin phosphodiesterase 1 (SMPD1) after loading of the cells with oxLDL as one potential reason for the increased membrane ceramide levels. SMPD1 is one of the key enzymes for the conversion of sphingomyelin to ceramide. We therefore hypothesized that not only increased expression but also increased cell surface SMPD1 could be responsible for this strong increase in membrane ceramides. To test this hypothesis SMPD1 protein localization was analyzed by confocal microscopy in MCSF treated, eLDL and oxLDL loaded macrophages (Fig 4). As anticipated oxLDL induced significantly higher cell surface ceramide fluorescence compared to control MCSF differentiation and loading with eLDL (Fig 4A). We additionally observed co-localization of ceramide with SMPD1 staining.

### oxLDL induces microdomains enriched in cholesterol and ceramides

**Fig 5:**
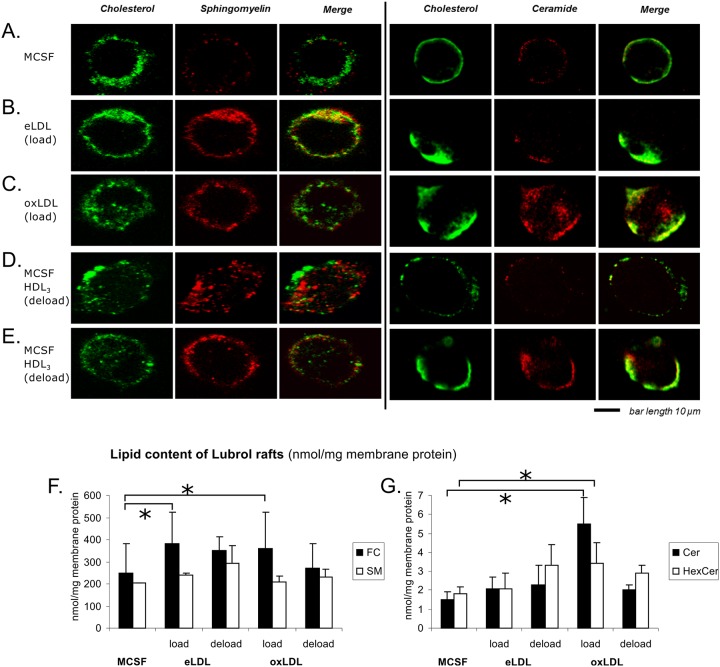
eLDL promotes formation of cholesterol/sphingomyelin rich membrane microdomains while oxLDL induces cholesterol/ceramide microdomains. **(A)** MCSF differentiation, **(B)** eLDL loading, **(C)** oxLDL loading, **(D)** eLDL loading with subsequent HDL_3_-deloading and **(E)** oxLDL loading with subsequent HDL_3_-deloading. Theta toxin was used for cholesterol and lysenin for sphingomyelin labeling. MID15B4 antibody was used for ceramide. Representative cells are shown.

The surface distribution of cholesterol, sphingomyelin and ceramide was furthermore visualized by confocal microscopy (Fig 5). In addition to the anti-ceramide antibody clone MID15B4, theta toxin was used for the staining of cholesterol and lysenin for sphingomyelin labeling. Loading with eLDL and oxLDL induced a stronger staining of sphingomyelin compared to MCSF differentiated control cells. Compared to MCSF differentiated control cells, the amount and distribution of cholesterol was similar after eLDL and oxLDL loading. MID15B4 antibody staining showed that macrophages loaded with oxLDL presented the highest amount of ceramide in the membrane, indicating the formation of microdomains enriched in ceramide.

Visually the staining patterns of sphingomyelin and ceramide were consistent with membrane microdomains. The most widely used criterion to substantiate microdomain association is based on insolubility with detergents. We therefore investigated isolated Lubrol detergent resistant membranes (DRM) with ESI-tandem mass spectrometry. Under these conditions cholesterol based membrane domains are preserved. As shown in Fig 5F and 5G and Table 1, treatment with eLDL and oxLDL led to an induction of cholesterol rich DRMs compared to MCSF differentiation. Ceramide (Cer) and also hexosylceramide (HexCer) content of Lubrol DRMs was only significantly induced during oxLDL loading but not during eLDL loading. This is an indication for the specific formation of ceramide-rich membrane microdomains upon oxLDL loading. Elevations of sphingomyelin were generally less prominent and did not increase significantly for either treatment.

**Table 1:** Quantitative determination of the lipid content of Lubrol rafts.

	FC	SM	Cer	HexCer
**MCSF (diff)**	247 +/- 136	206 +/- 10	1.5 +/- 0.4	1.8 +/- 0.4
**eLDL (load)**	383 +/- 142 *	238 +/- 11	2.1 +/- 0.6	2.1 +/- 0.8
**eLDL (deload)**	349 +/- 66	295 +/- 77	2.3 +/- 1.0	3.3 +/- 1.1
**oxLDL (load)**	358 +/- 168 *	208 +/- 28	5.5 +/- 1.4 *	3.4 +/- 1.1 *
**oxLDL (deload)**	272 +/- 111	231 +/- 37	2.0 +/- 0.3	2.9 +/- 0.4

Free cholesterol (FC), sphingomyelin (SM), ceramide (Cer), hexosylceramice (HexCer). Values are given in nmol/mg protein.

* p<0.05 compared to MCSF using Student’s t-test. n = 3

A co-localization of cholesterol and sphingomyelin could be observed mainly in cells loaded with eLDL and only to a lesser extent in oxLDL treated cells. This co-localization was abolished when the cells were deloaded with HDL_3_ indicating a disruption of the raft structure. In contrast oxLDL loaded cells showed a large amount of cell surface domains rich in cholesterol and ceramides. Interestingly this co-localization of ceramide and cholesterol was not disrupted by HDL_3_ mediated deloading.

Further evidence for the formation of membrane microdomains with a different lipid composition after loading with eLDL and oxLDL was obtained by stimulated emission depletion (STED) nanomicroscopy in collaboration with Prof Hell/PD Dr Harke at the MPI in Göttingen. For this purpose an antibody against FcγRIIA (CD32) was used to selectively stain membrane microdomains. This method revealed a cluster size of ceramide enriched microdomains between 30 and 50nm with a tendency of enlargement upon lipid loading compared to MCSF differentiation (S5 Fig).

### ApoE binding to oxLDL treated cells is facilitated

**Fig 6:**
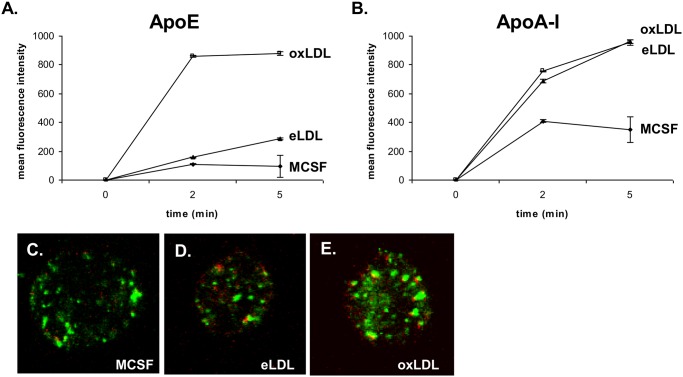
Binding and uptake of apoE and apoA-I determined by flow cytometry and confocal microsopy. 4°C binding of fluorescent labeled apoE (Cy3-apoE) **(A)** and apoA-I (Cy5-apoA-I) **(B)**. Flow cytometry was performed on control macrophages as well as on lipid loaded cells after 2 and 5 min in three independent different experiments. Additionally using confocal microscopy, binding of fluorescent labeled apoE (Cy3-apoE, green staining) and apoA-I (Cy5-apoA-I, red staining) was visualized following M-CSF differentiation **(C)** as well as after eLDL **(D)** and oxLDL (E) loading.

As described previously by Morita et al [29], apoE preferentially binds to ceramide enriched domains compared to sphingomyelin-enriched domains on emulsion particle surfaces. We therefore analyzed 4°C binding uptake of fluorescent labelled apoE (Cy3-apoE) and apoA-I (Cy5-apoA-I) in MCSF differentiated, eLDL and oxLDL loaded macrophages by flow cytometry and confocal microscopy. Flow cytometry revealed a strongly enhanced binding of apoE to oxLDL loaded cells (Fig 6A) consistent with increased levels of surface ceramides. In contrast to apoE, apoA-I exhibited a comparable binding capacity at 4°C to eLDL and oxLDL loaded cells (Fig 6B). Confocal microsopy likewise showed a stronger fluorescence of Cy3-labeled apoE (green fluorescence) with oxLDL loaded cells compared to control and eLDL treated cells (Fig 6C–6E). Using Cy5-apoA-I a generally weaker staining was observed.

## Discussion

Phagocytic differentiation had only minor effects on the cellular sphingolipid composition. Levels of total sphingomyelin and total ceramides remained unchanged, while free cholesterol as the major determinant of membrane fluidity decreased. This altered membrane fluidity most likely prepares the cell for its phagocytic function [51].

In contrast, treatment with free cholesterol-rich eLDL particles promoted foam cell formation while loading with oxLDL predominantly provoked endo-lysosomal cholesterol accumulation with characteristics of phospholipidosis most likely due to inhibition of lysosomal hydrolases and release of cholesterol from these organelles [52]. As a result oxLDL leads to a dysfunction of vesicular trafficking with endolysosomal phospholipidosis and impaired cholesterol efflux [10, 12, 14, 32]. All further measurements were interpreted based on the assumption that lipids from eLDL and oxLDL particles are taken up about equally. A higher concentration of oxLDL was used because the cholesterol loading efficiency of eLDL is excessively higher as compared to oxLDL [53]. Therefore, to achieve a significant oxLDL dependent cholesterol effect we used a lower concentration of eLDL and the same concentration of oxLDL as in other publications (e.g. [54, 55]).

eLDL induced 54-fold higher levels of esterified cholesterol compared to unloaded control cells, while oxLDL showed only a 4.4-fold increase of CE species. Interestingly oxLDL increased the CE species 18:1 more than expected from the total increase. Free cholesterol was also elevated stronger by treatment with eLDL than by oxLDL but to a generally lower extent (2.5-fold and 1.8-fold respectively). These changes are in accordance with previously published data showing that eLDL due to the treatment with cholesterylester-hydrolase (acid lipase) contained a higher amount of FC at the expense of CE compared to LDL and oxLDL [56].

In contrast oxLDL loading of macrophages led to an upregulation of cellular ceramide content. On the species level, ceramides 16:0, 18:0, 20:0, 22:0, 23:0, 24:1 and 24:0 increased significantly around 3.7-fold. Likewise the specific cell surface ceramide composition at the plasma membrane was affected as shown by flow cytometry and fluorescence microscopy. On the other hand total cellular sphingomyelin content remained constant after lipoprotein loading, while plasma membrane sphingomyelin was increased after both eLDL and oxLDL lipoprotein loading.

eLDL and oxLDL particles have a comparable ceramide and sphingomyelin composition, therefore the increase in ceramides cannot be attributed to differential uptake of sphingolipids from the lipoprotein [56]. An endogenous source of ceramides in a mammalian cell is the sphingomyelin-ceramide rheostat. Ceramide can be released from complex sphingolipids leading to increases in ceramide and decreases in sphingolipid levels. Previously it has been shown that oxLDL can induce this mechanism by stimulation of neutral and acid SMase [57]. Oxysterols as exclusive components of oxLDL could additionally contribute to the elevation of cellular ceramide levels [58]. Our experiments are consistent with these observations and demonstrate that the mRNA expression of acid SMase, neutral SMase and neutral SMase activation associated factor is stronger upregulated during oxLDL compared to eLDL loading. Therefore the transcriptomic basis for an activation of the salvage pathway and not *de novo* synthesis as the reason for the increased ceramide levels after loading with oxLDL could be shown experimentally within the scope of this study. Naturally in the complex cellular environment, subsequent protein levels and further regulation of enzyme activity by additional factors such as gluthathion need to be considered.

oxLDL is taken up via scavenger receptors CD36, SR-A, SR-B, LOX-1 into early endosomes and in contrast to eLDL not stored in lipid droplets but exerts direct effects on transcription [10]. Exogenous sphingolipid can then be processed to ceramide and induce caspases and PLA_2_. oxLDL effects are also, at least in part, mediated through the transcription factors PPAR-γ and NF-κB. Various bioactive oxidized lipids in oxLDL, such as 9-HODE and 13-HODE, are known to trigger NF-κB activation [59]. Likewise, oxLDL has a much higher content of Lysophosphatidylcholine (Lyso-PC) in comparison to unmodified LDL [56]. Lyso-PC can stimulate protein kinase C and tyrosine kinases leading to subsequent NF-κB activation [60]. Also bioactive oxysterols (especially 7-keto, 7-hydroxy and 25-hydroxycholesterol) are present in oxLDL [61]

Using confocal microscopy we found an accumulation of SMPD1 at the cell surface. SMPD1 is usually found in the endolysosomal compartment, but translocation to sphingolipid rich membrane microdomains converting them into ceramide rich microdomains that facilitate receptor clustering is well established [62–64]. Sudden increases in ceramide levels due to the action of plasma membrane associated SMase may have an important impact on macrophage cholesterol homeostasis, bacterial host defense, and apoptosis [65].

Besides increased ceramide production after oxLDL loading we provide data that oxLDL induced microdomains are enriched in cholesterol and ceramides while microdomains in cells treated with eLDL have higher levels of cholesterol. Sphingomyelin was not increased significantly in Lubrol rafts from eLDL loaded cells, only a tendency was visible. Ceramide associates strongly with lipid rafts and stabilizes them. This can induce the formation of unusually large membrane domains in plasma membranes detectable by the monoclonal ceramide antibody which we used for our experiments [62]. In studies using lipid vesicles it was found that natural and saturated synthetic ceramides displace cholesterol from lipid microdomains [66]. In our macrophage experiments we detected a co-localization of cholesterol and ceramide mainly during oxLDL loading indicative for the coexistence of cholesterol and ceramide in membrane microdomains. In collaboration with Prof Hell/PD Harke, MPI Göttingen, we have furthermore performed pilot experiments with STED-nanomicroscopy which allows a spatial resolution of 30–70 nm. In our experiments we used a staining with an anti-FcγRIIa (CD32) antibody to visualize lipid rafts. CD32 is a polymorphic transmembrane glycoprotein that is characteristically associated with cholesterol-rich membrane subdomains [67]. Using this method we found a ceramide membrane microdomain size between 30 and 50 nm with a tendency to enlarge upon lipid loading.

oxLDL also exerts oxidative stress on the macrophage membrane. Previous experiments using H_2_O_2_ as a stimulus could demonstrate an increase in the number, but not the size, of membrane microdomains, therefore an involvement of survival pathways was assumed [68]. Moreover the major oxysterol in oxLDL, 7-ketocholesterol is able to activate src kinase [69] by cholesterol depletion and raft disruption [70].

In addition to the higher buildup of cell surface ceramide during oxLDL loading compared to eLDL loading, an increase of ceramide derived glycosphingolipids and GM1 ganglioside as detected by cholera toxin was measurable. This could reflect either increased net synthesis from ceramide or enhanced translocation from the Golgi to the plasma membrane during oxLDL loading. Alternatively, impaired degradation of glycosphingolipids or decreased endocytosis could also play a role. A little care needs to be taken in interpreting the staining of GM1 using cholera toxin. Although this method is widely used and GM1 represents the most efficient ligand, binding of cholera toxin to other glycosphingolipids, such as GD1b, fucosyl-GM1, B-GM1, GM2, GT1b, GD1a, GM3 and asiolo-GM1 have been reported in the literature and cannot be ruled out [71–75].

As a physiological consequence of the altered surface expression of ceramide and the differential membrane lipid microdomain formation during eLDL and oxLDL loading we demonstrated a stronger affinity of apoE to cells treated with oxLDL as compared to eLDL treated cells. Since ApoE staining was not only observable at the cell membrane also internalization of the lipoprotein seems reasonable to assume. Furthermore a more pronounced fluorescence in oxLDL treated cells is consistent with the observation that apoE preferentially binds to ceramide enriched membrane domains of emulsion particles treated with sphingomyelinase and not to sphingomyelin emulsion particles [29]. It is also compatible with the interaction of apoE and ceramides during nerve regeneration and remyelination [30]. ApoA-I binding however, was identical between eLDL and oxLDL loaded macrophages. This indicates that binding of extracellular apoA-I does not depend on microdomain ceramide content and may rather correlate with membrane cholesterol [76].

Endogenously synthesized ceramide and sphingomyelin enhances cellular cholesterol efflux to apoA-I by increasing the presence of ABCA1 at the cell surface [77]. This leads to a block of the transport of CD36 to the plasma membrane preventing further uptake of oxLDL [78]. In our experiments we detected a strong induction of ABCA1 mRNA and protein in lipoprotein loaded cells. Especially loading with oxLDL induced a considerable increase in cellular ABCA1, consistent with endolysosomal storage of oxLDL and an expansion of the trans-Golgi network. Treatment with apoAI also has the potential to increase caveolar cholesterol by inducing a stronger mobilization from intracellular sources than promoting its efflux [79]. ABCA1 in macrophages, by reducing membrane cholesterol has been described to reduce toll-like receptor trafficking to lipid rafts and in this way moderate inflammation [80]. In contrast ABCA1 deficient macrophages have enlarged, cholesterol rich lipid microdomains with higher levels of TLR4 and an increased reactivity to lipopolysaccharides [81].

ApoE respectively apoE-rich lipoproteins such as VLDL, chylomicron remnants and HDL_1_ also contribute to presentation of lipid antigens by high affinity binding of endogenous or exogenous antigens mainly ceramide derived glycosphingolipids [82]. Therefore higher apoE binding to ceramide rich membrane microdomains of oxLDL loaded macrophages may also promote antigen presentation and immune modulation.

## Conclusions

In summary, we could demonstrate that the two atherogenic lipoprotein modifications eLDL and oxLDL induce two different types of lipid membrane microdomains in human macrophages. These changes are caused most likely by an induction of sphingomyelinases due to an activation of the salvage pathway as well as an increased translocation of acid sphingomyelinase to the plasma membrane. The formation of cholesterol/ceramide rich microdomains during oxLDL loading leads to a higher cell surface binding of apoE in contrast to the uniform affinity of apoA-I which could contribute to impaired lipid efflux and enhancement of inflammatory processes. This supports the view that differential formation of lipid membrane microdomains in macrophages could lead to differential functional responses towards atherogenesis.

## Supporting Information

S1 Fig Quantitative mass spectrometric analysis of individual lipid species during differentiation and loading. Lipid species content of extravasated blood monocytes was analyzed after four days phagocytic differentiation (A-D) or after 24 h lipoprotein loading (E-H) with eLDL or oxLDL, respectively. SM annotation is based on the assumption that sphingosine d18:1 is present. Mean +/- SD. * = p< 0.05. n = 6.
(PDF)

S2 Fig Relative gene expression and protein levels of (A) ABCA1, (B) ABCG1 and (C) PLTP. Gene expression was analyzed using TaqMan RT-PCR analysis. Results are presented as mean +/- SD. Protein levels were assessed using Western Blot analysis with appropriate antibodies. Reference protein expression of ATP-Synthase is depicted in (D). n = 3.
(PDF)

S3 Fig Ceramide synthesis and degradation pathways. (PDF)

S4 Fig Levels of sphingosine during monocyte to macrophage differentiation and after loading with lipoproteins. (PDF)

S5 Fig Determination of CD32 labeled membrane microdomain sizes by STED microscopy. (PDF)
