# Supplementary figures and images for: oxLDL and eLDL Induced Membrane Microdomains in Human Macrophages
Source: PLoS One. 2016 Nov 21;11(11):e0166798. doi: 10.1371/journal.pone.0166798 (PMC5117723; doi:10.1371/journal.pone.0166798)

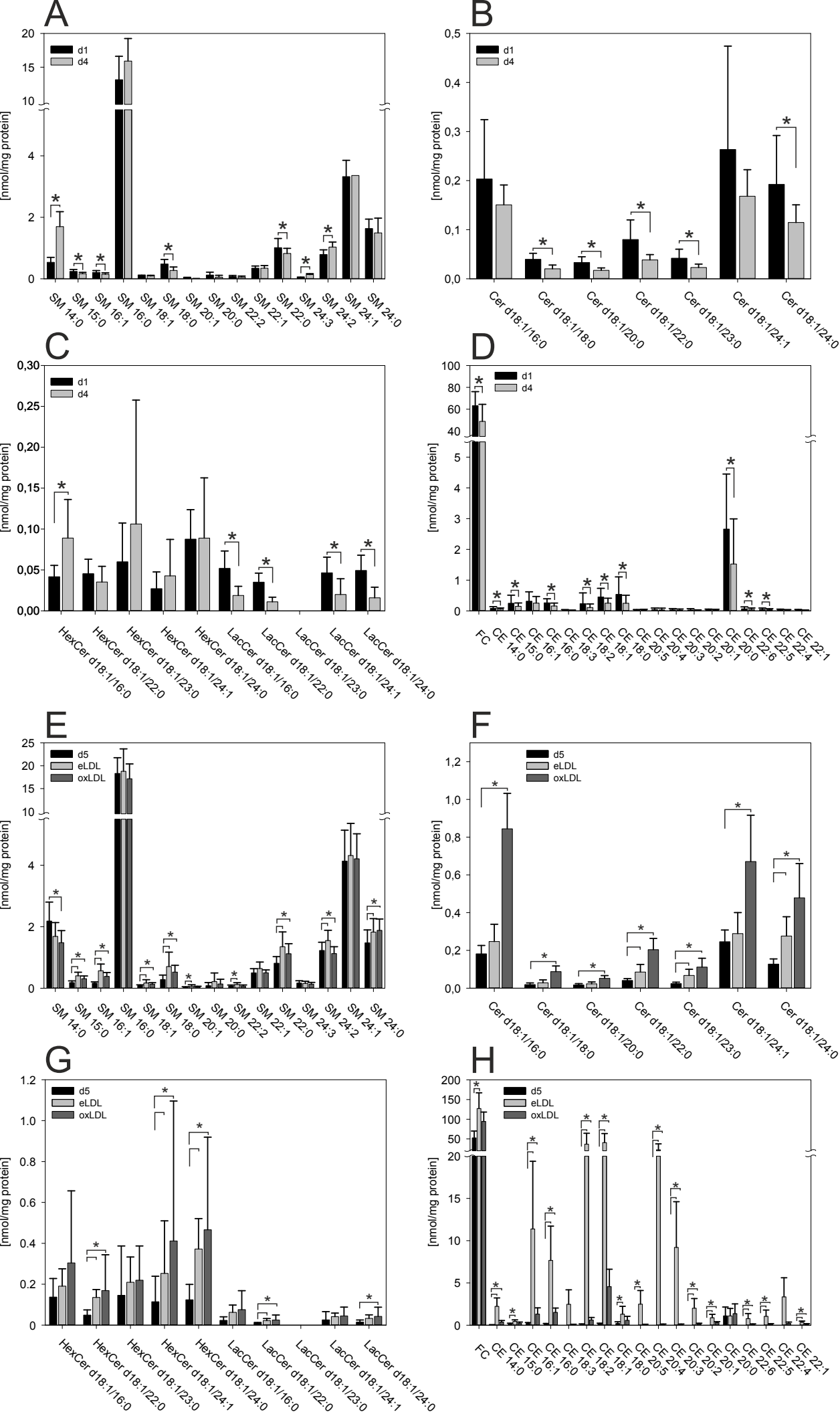

Supplement: S1 Fig — Lipid species content of extravasated blood monocytes was analyzed after four days phagocytic differentiation (A-D) or after 24 h lipoprotein loading (E-H) with eLDL or oxLDL, respectively. SM annotation is based on the assumption that sphingosine d18:1 is present. Mean +/- SD. * = p< 0.05. n = 6. (PDF) [file pone.0166798.s001.pdf]

A mRNA

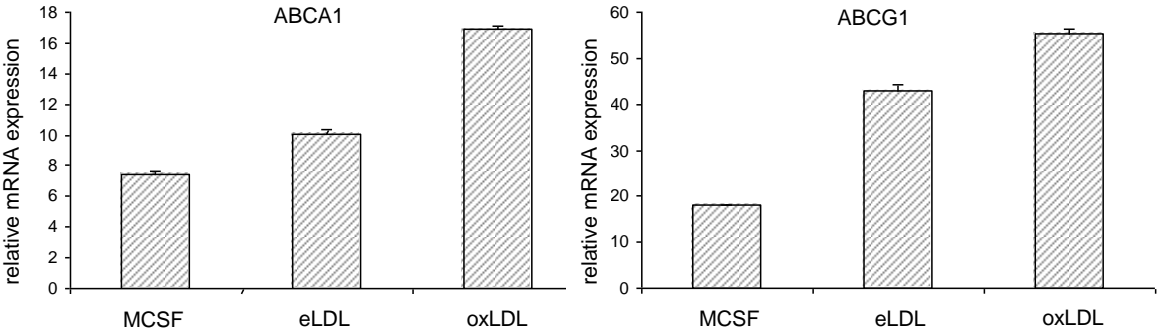

B protein

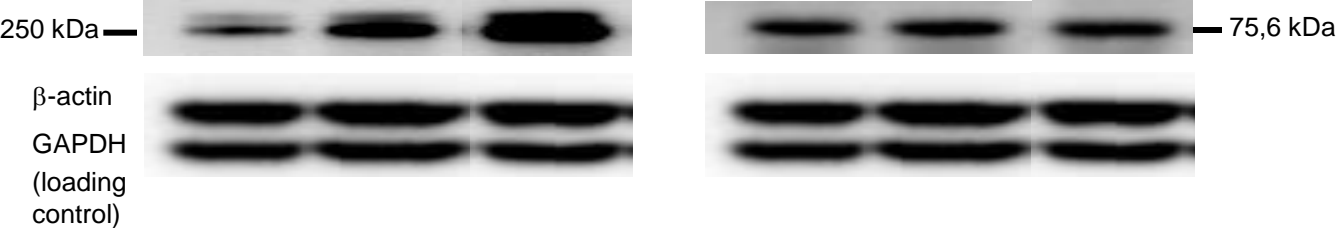

Supplement: S2 Fig — Gene expression was analyzed using TaqMan RT-PCR analysis. Results are presented as mean +/- SD. Protein levels were assessed using Western Blot analysis with appropriate antibodies. Reference protein expression of ATP-Synthase is depicted in (D). n = 3. (PDF) [file pone.0166798.s002.pdf]

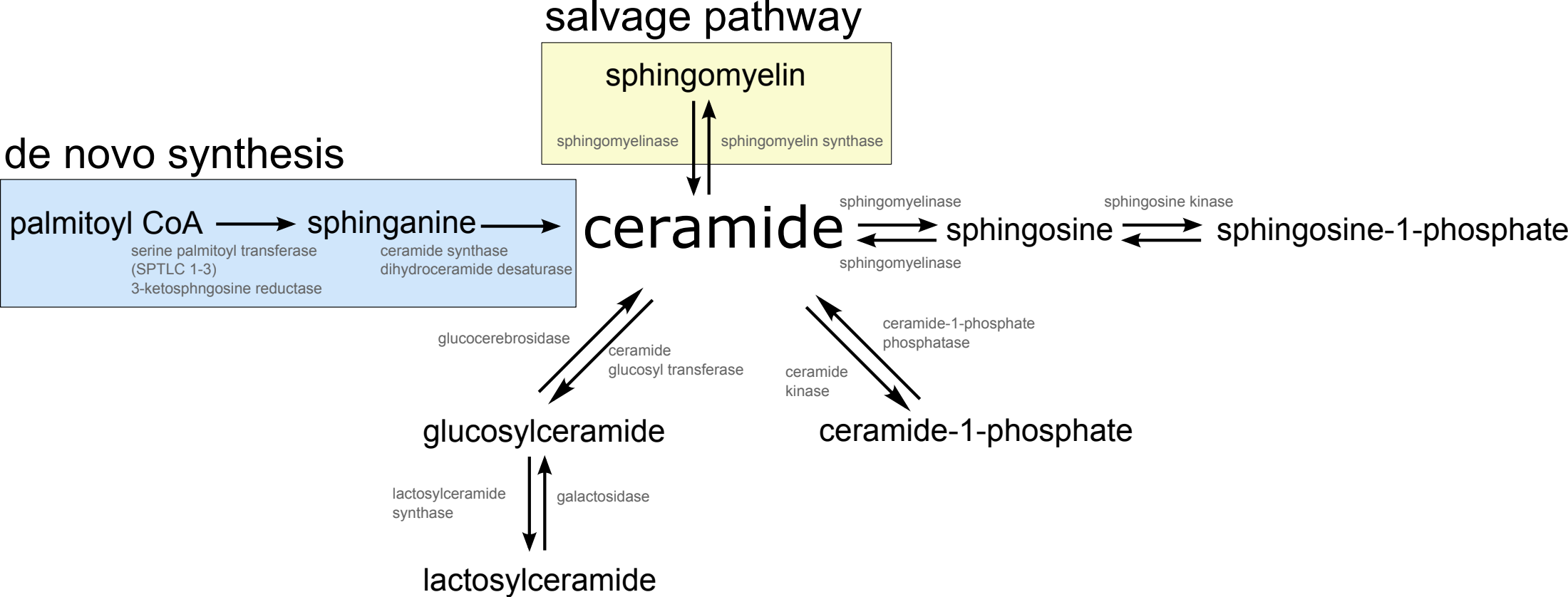

Supplement: S3 Fig — (PDF) [file pone.0166798.s003.pdf]

# sphingosine

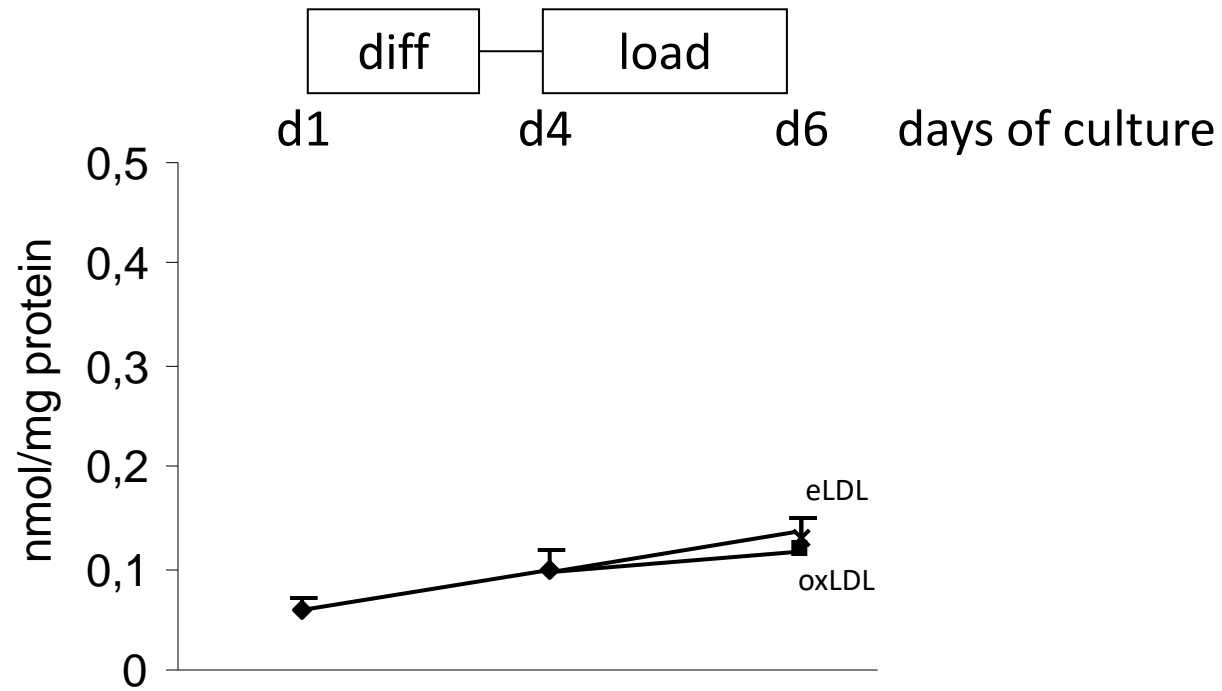

Supplement: S4 Fig — (PDF) [file pone.0166798.s004.pdf]

## MCSF

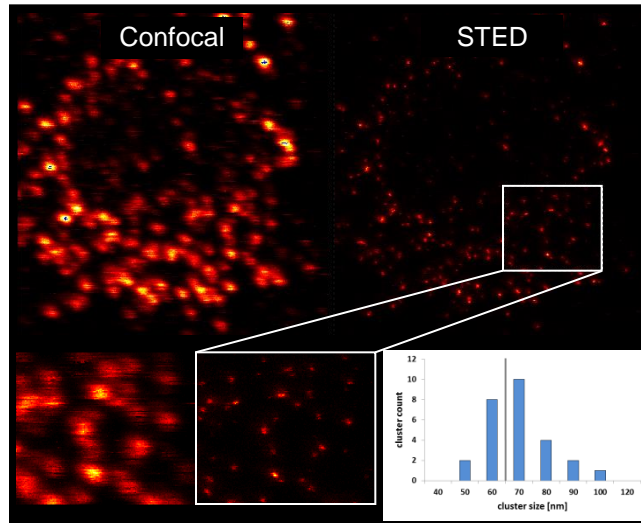

## eLDL

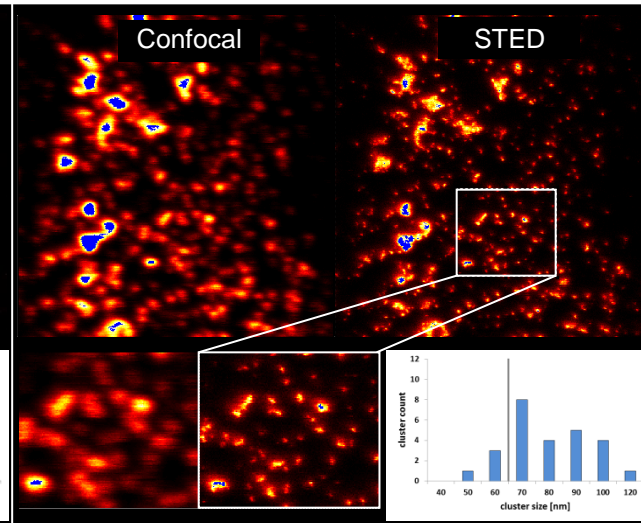

## oxLDL

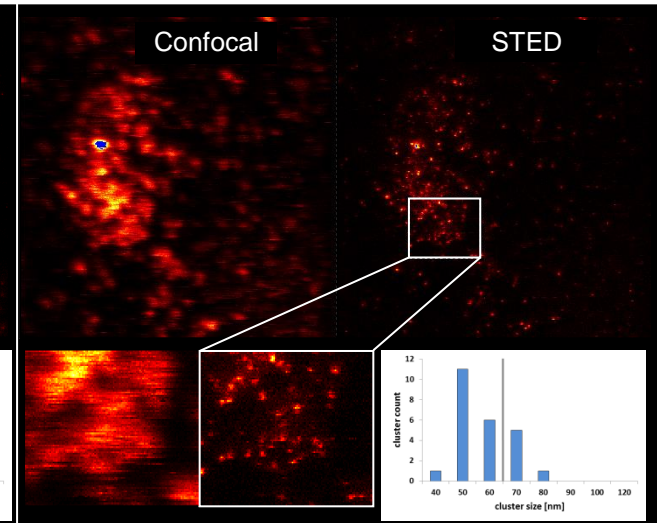

Supplement: S5 Fig — (PDF) [file pone.0166798.s005.pdf]
